# The Observer’s Lens: The Impact of Personality Traits and Gaze on Facial Impression Inferences

**DOI:** 10.16910/jemr.17.3.5

**Published:** 2024-08-19

**Authors:** Kuangzhe Xu, Toshihiko Matsuka

**Affiliations:** Institute for Promotion of Higher Education, Hirosaki Unibversity, Japan; Department of Cognitive and Information Science, Chiba University, Japan

**Keywords:** Eye movement, Personality Traits, Impression Inference, First impressions, Bayesian Statistics

## Abstract

Previous studies on facial impression inference have focused on the physical features of faces, with only a few considering the effects of the observer. This study explored how participants’ personality traits directly and indirectly affect the impression inference of human faces. Specifically, we examined how observers’ personality traits impact their eye movements, which in turn influence impression inferences. Experiment 1 found relationships between participants’ personality traits and eye movements, but these did not significantly impact impression inferences. In Experiment 2, we manipulated observers’ observational behavior to control for the potential interactive effect between facial features and participants’ eye movements during impression inference. This manipulation suggested that focusing on different areas of faces leads to different impression inferences. It also suggests that the same person might have different impressions of the exact same face by changing their observational behavior. These results deepen our understanding of the impact of facial features and participants’ personality traits on impression inferences, indicating that observers’ personality traits and observational behavior play a significant role in impression formation.

## Introduction

First impressions of faces can have strong societal impacts, even though they are often subjective and do not reflect a person’s actual personality. Therefore, research on human faces has been actively conducted for a long time. Among these studies, one of the most extensively researched topics is how faces are recognized and the outcomes of these recognitions. For example, Groner (16) showed that similarity judgments of human faces can be represented by twelve dimensions, some of them representing structural anatomical features, and others related to affective judgments. Additionally, evolutionary preferences suggest that individuals with attractive faces are more likely to be chosen as spouses (23; 35; 69). Similarly, some studies have shown that attractive faces are recognized faster than less attractive ones, and people tend to look at more attractive faces for longer durations (36; 43; 1; 10).

Building on these insights, individuals often utilize others’ facial features to predict and judge other people’s attractiveness, personality traits, and other characteristics. For instance, it has been substantiated that assessing a person’s personality from their facial appearance accurately is feasible (29; 54; 58). Additionally, studies have explored how individuals decide on their subsequent actions based on the information gleaned from the faces of people they encounter for the first time (60; 24; 20; 9). Furthermore, gender differences in evaluating attractiveness and cuteness across various facial types (adults and infants) have been identified (17; 59). Moreover, research conducted by those perceived as competent scientists is often rated as high-quality (14), and the impact of skin conditions on facial attractiveness has been scrutinized (21). Perceptions of individuals in social and professional activities, such as deciding whom to vote for (“trustworthy” face), job searching (“capable-of-working” face), and assessing business acumen (“competent” face), are also strongly influenced by facial features (18; 40; 28; 56; 27; 37; 55; 44). Recently, studies analyzing facial features using advanced techniques, such as computational models and machine learning, have partially explained the relationship between the physical features of faces and impression judgments (38; 57; 32; 5).

The majority of studies examined how the physical properties (e.g., lip thickness, nose width) affects first impressions. This series of research is based on the implicit assumption that faces with specific features uniformly convey certain impressions to observers, regardless of the observers’ characteristics. Differences among observers are considered errors, and these individual differences are excluded from the analysis. However, features of the perceived also play a role. Previous research focusing on observer characteristics has primarily dealt with factors such as age (2), gender (46), occupation (50; 3), race (53), or health issues (22; 4). For example, some studies focusing on the age characteristics of observers suggested that children and adults have different impressions of the same face (11; 12). In the present study, we examine the role of personality traits of the observer on first impressions of others. In other domains, such as Behavioral Economics and Education, personality traits have been shown to affect impressions from observers, and it can therefore be expected that personality traits will also affect first impression of faces.

This paper attempts to provide additional evidence on how observers’ characteristics, particularly personality traits, affect the inference of facial impressions by exploring both the direct and indirect effects of observers’ personality traits on impression inferences. The direct effect implies that observers’ personalities influence facial impression ratings, as suggested by previous research. For instance, experiments using a large number of facial images showed that most of the impression inference ratings were associated with the characteristics of the observers (19). Furthermore, research suggests that personality traits have a significant impact on a person’s cognitive formation (51, 52). We hypothesize that personality traits, among various observer characteristics, play a crucial role in impression inferences.

When studying the effects of personality traits on first impressions, it is important to also monitor observers’ eye movements, as previous research has suggested that eye tracking data can reveal which facial features are most attended to and how these features influence the observer’s overall impression (13; 61). For instance, Asians generally focus on the center of the face, whereas Caucasians tend to look more frequently at the eyes and mouth compared to Asians (7; 34). On the other hand, studies by Xu (63) have shown that the impact of personality traits, which can more directly represent individual characteristics than broad definitions like culture, cannot be ignored. Furthermore, subsequent studies have investigated the relationship between eye movements and personality traits when solving facial type recognition tasks (45; 68, 67), and others have attempted to predict personality traits using eye movements (6). These studies demonstrated that observers with different personalities look at others’ faces differently; for example, more extroverted individuals tend to look at the eyes and mouth more frequently than less extroverted individuals (64; 65). Collectively, these results suggest that different people may look at the same face in different ways. When a face is observed differently (e.g., when only limited areas are focused on), the very same face may be perceived differently or result in different impressions. This research examines the potential indirect effect of observers’ personality traits on impression inferences.

Most previous studies on facial impressions have regarded individual differences among observers as variations. However, this paper takes an opposite perspective, considering that individual differences among observers also play a role in impression inferences. Therefore, we hypothesized that personality traits have both direct and indirect effects on impression inferences. Indeed, we conducted experiments to test this hypothesis by investigating the relationship between observer characteristics and facial impression inferences. Specifically, we assumed that observers’ personality traits influence their different observational behaviors towards faces, and these behaviors, in turn, affect facial impression inferences. In this study, we applied original processing to the eye movement data recorded during the experiment to consider the effects of peripheral vision. This processed data was used in the analysis as a measure of the participants’ observational behavior. For data analysis, we employed hierarchical Bayesian models to quantitatively examine how personality traits and observational behavior interactively influence various facial impression inferences.

## General Methods

### Impression inferences

In the present experiment, we used one of the most well-known sets of personality traits, namely Big Five personality traits, in line with previous studies (29; 63). The five personality traits were Agreeableness, Conscientiousness, Extraversion, Neuroticism, and Openness to experience. The five factors were translated into Japanese according to previous studies (39). The actual Japanese words used in the present research are available in Supplementary Materials (https://osf.io/bn93u). For those who were unfamiliar or uncertain about those personality traits, a brief explanation of the five factors was also given verbally before the experiment began. During the experiment, the experimenters did not intervene at all, leaving the decisions to the participants.

### Stimuli

We used 50 pictures (25 male and 25 female) of East Asian faces from Hong Kong University’s database (26). All pictures were taken from the front without any emotional expressions. The pictures were converted to black and white with Photoshop and the brightness across all pictures was set to be identical. The pictures were cropped to 412 × 558 pixels and adjusted so that both eyes were at the same height. In order to avoid memory effects and other unwanted effects, each picture appeared exactly once in the present experiment, following previous research (66; 62). Each personality trait (described below) was rated with a set of 10 pictures that did not appear in other inference tasks. The face stimuli were pre-assigned randomly to each impression task. In so doing, we randomly divided the 25 male facial stimuli into five equally numbered groups, and then each stimuli group was randomly assigned to one impression inference task (e.g., inferring faces’ Agreeableness). The same processes were applied to the 25 female stimuli, resulting in 10 stimuli (five male and five female faces) for each task. The sets were fixed for all participants, and thus all participants rated the same set of facial stimuli for each impression inference task. Note that, in model building, we incorporated random effects of faces (e.g., particular faces are more likely to be seen on the eyes or are rated higher on Openness). This should eliminate effects due to arbitrary stimulus allocation (see ‘Model Building’ session below).

### Apparatus

In this study, we used a Tobii T-120 monitor-mounted eye tracker (1024 × 768 pixels resolution) to record stimuli images and eye movements. The experiment was conducted and controlled using the Tobii Pro SDK Python API and PsychoPy software. They were synchronized with the eye tracker to ensure that eye movements and stimulus images were correctly recorded simultaneously. As per previous research, participants rested their chin in a chin rest at a distance of 80 cm from the monitor to reproduce an interpersonal environment at a distance of 65 cm.

### Data preprocessing

**Figure 1.**
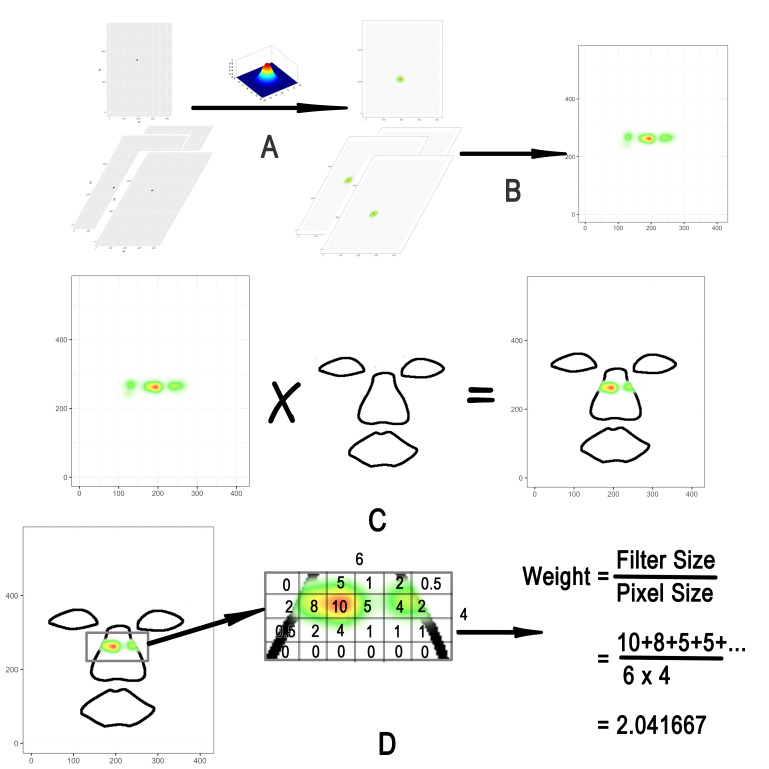
Data preprocessing method. Note. A. The Gaussian filter (sd. = 10) was applied to the recorded gaze data every 1 Hz. B. The filtered data within the same session were superimposed, and the weight of the gaze data was then calculated. C. An individualized face mask was applied to the weight data to extract the weight of each area. D. The weight of each area was divided by the size of the corresponding area (pixels count), and the weight of the one-pixel unit for each part was calculated. (The above numbers are examples, not actual results).

In order to have reliable data, we excluded data where eye-tracking sampling rates were less than 60%. This 60% sampling rate refers specifically to the sampling rate for a single stimulus, not the overall rate. That left us 34, 33, 34, 32, and 33 participants for Agreeableness, Conscientiousness, Extraversion, Neurosis, and Openness inference tasks, respectively. Each of these exceeds the minimum experimental sample size of 30 participants as indicated by previous research Smith et al. (48). To construct a Bayesian generalized linear mixed model, we preprocessed the eye-tracking data. The detailed processing is shown in Figure 1. First, based on the previous research (8), we applied a Gaussian filter with a 10-pixel standard deviation to every gaze data (A). The filtered data within a single session were superimposed, and the weight of the gaze data was then calculated (B). For all stimuli, a mask for three different areas of the face, namely eyes, nose, and mouth, was drawn by hand using Intuos Pro PTH-660. One mask corresponded to one facial image. The extent of each part’s contour was determined at the author’s discretion.

An individualized face mask was applied to the weight data to exclude any data outside of the areas of interest. In the present study, we only extracted weight data that reside within the eyes, nose, and mouth (C). This is because many previous studies commonly considered the eyes, nose, and mouth to be important and critical features of faces (7; 42; 63). The weight of each facial part was divided by the size (pixel counts) of the corresponding area to equate differences in the size of the areas of interest. Subsequently, a model analysis was performed using these preprocessed data.

### Data analysis

All data analyses in this study were conducted using Bayesian statistical models. There are two main reasons for using Bayesian statistics. First, Bayesian statistics can flexibly accommodate complex distributions, making it an ideal method for analyzing intricate data like eye movements. Second, Bayesian statistics allow us to estimate posterior distributions based on data sampling, enabling a more accurate understanding of population characteristics.

**Figure 2.**
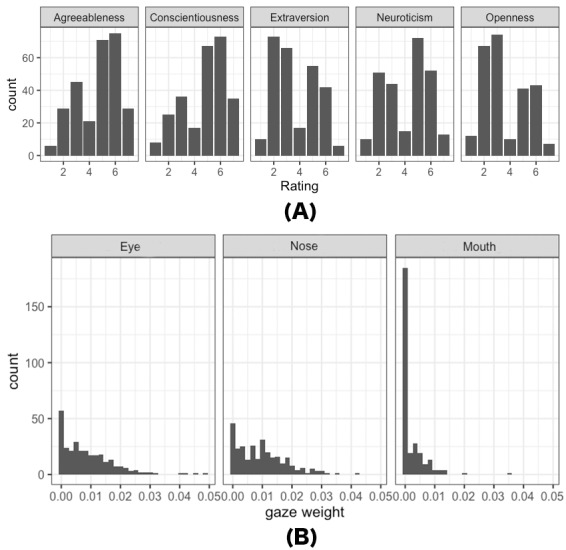
(A) Distribution of participants’ rating scores for the five impressions in Experiment 1, and (B) distribution of eye movements of all participants (N=34) in Experiment 1 when rating extraversion for all stimuli (N=50). Refer to the Data Preprocessing section for the method of calculating gaze weight. The higher the gaze weight, the more attention is paid to the corresponding area.

Figure 2 (A) displays the distributions of impression inference ratings for all participants. Similarly, Figure 2 (B) illustrates the distributions of eye movements (specifically, gaze weight) for all participants when inferring the trait of Extraversion, serving as an illustrative example. Since the primary objective variables in our analysis exhibit distributions that are closer to a discrete shape, an ordinal logistic regression model was used. The latter distribution, representing another set of objective variables, appears as a mixed distribution characterized by many zeros and a highly skewed distribution. For fitting highly skewed variables, beta regression is recommended. Therefore, we consider this mixed distribution to follow the zero-inflated beta distribution (ZIB) (70; 66; 62).

**Figure 3.**
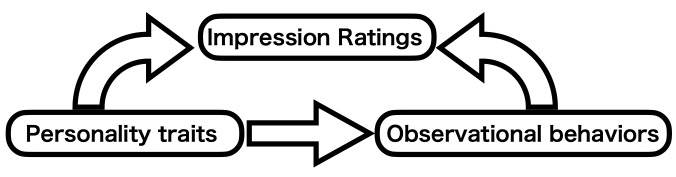
Simple relationship diagram illustrating how participant characteristics and impression inference ratings are connected during impression inference.

This experiment aimed to examine how participants’ characteristics (personality traits and observational behavior) influence impression inferences. We developed and fitted a model described in Figure 3. Our model considers that the participants’ personality traits have two routes to affect facial impression inference: a direct effect and an indirect effect through eye movements. That is, the participants’ personality traits affect how they observe others’ faces, which in turn affects how they infer impressions of the faces. Bayesian estimations were performed to see what sort of relationships among the variables exist and the validity of the model.

### Model building

The zero-inflated mixed beta regression (ZIB) model assumes that the objective variable comes from two distributions, namely the Bernoulli distribution and the Beta distribution. The Bernoulli distribution is associated with whether participants look at the areas of interest at least once or not. The Beta distribution is associated with how much participants look at the area of interest.

Equations (1) through (6) indicate our ZIB model describing the relationship between participants’ personality traits and eye movements for a particular area ( k), where i and j are indices for participants and stimuli, respectively. More specifically, Equations (1) and (2) model the observational behavior ( $G_{ijk}$) which corresponds to if area k was looked at least once by observer i for face j, and if they did, how much the area was looked at. “Whether or not the area of interest was looked at” was modeled using the Bernoulli distribution ( $q_{ijk}$), and implemented as a logistic regression model. “How much the area of interest was looked at” was modeled using the beta distribution ( $a_{ijk}$, $b_{ijk}$). The parameters for the beta distributions were further modeled as shown in Equations (4) and (5). The linear parts of our model indicate that the participants’ personality traits ( $p_{il}$) influence whether a particular area was looked at (Eq. (3)) and the extent to which the area was looked at (Eq. (6)). We treated these types of effects as fixed effects ( $\beta^{Z}$ for Bernuolli and $\beta^{B}$ for Beta), with the participants ( $r_{ik}^{Z}$ and $r_{ik}^{B}$) and facial stimuli ( $r_{jk}^{Z}$ and $r_{jk}^{B}$) as random effects. The model proposed in this study considers different observers have different tendencies for looking at particular areas of faces as well as how they infer impressions of faces above and beyond their personality traits. In addition, we believe different faces may attract different eye movements (e.g., a face having attractive eyes) or result in different impressions because of their facial features. For these reasons, we incorporated random intercept effects of participants and faces in our models.

(1) $$\begin{array}{c} G_{ijk}∼ZIB\left(q_{ijk},a_{ijk},b_{ijk}\right) \end{array}$$

(2) $$\begin{array}{c} ZIB\left(G_{ijk}|q_{ijk},a_{ijk},b_{ijk}\right)=\left\{ \begin{array}{cc} Bern\;\left(0\;|q_{ijk}\right)\;\;\;\;\;\;\;\;\;\;\;\;\;\;\;\;\;\;\;\;\;\;\;\;\;\;\;\;\;\;\;\;\;\;\;\;\;\;\;\;\;\;\;\;\;\;\; & \;\left(\;G_{ijk}\;=\;0\;\right)\\ Bern\;\left(1\;|q_{ijk}\right)\;\times Beta\;\left(G_{ijk}\;\right|a_{ijk},\;b_{ijk})\; & \;\left(\;G_{ijk}\;>\;0\;\right) \end{array}\right.\; \end{array}$$

(3) $$\begin{array}{c} q_{ijk}=\frac{1}{1+exp\left(-\left(\alpha_{k}^{Z}+\sum_{l=1}^{5}\beta_{kl}^{Z}p_{il}+r_{ik}^{Z}+r_{jk}^{Z}\right)\right)} \end{array}$$

(4) $$\begin{array}{c} a_{ijk}=\phi \cdot \mu_{ijk} \end{array}$$

(5) $$\begin{array}{c} b_{ijk}=\phi \left(1-\mu_{ijk}\right) \end{array}$$

(6) $$\begin{array}{c} \mu_{ijk}=\frac{1}{1+exp\left(-\left(\alpha_{k}^{B}+\sum_{l=1}^{5}\beta_{kl}^{B}p_{il}+r_{ik}^{B}+r_{jk}^{B}\right)\right)} \end{array}$$

Equations (7) through (8) i indicate our ordered logistic regression model for personality impression inferences. An ordered logistic regression model is a model where the objective variable is on an ordinal scale. There were two types of predictor variables in this model. One was the eye movements estimated in Equation (1) which were influenced by the participants’ personality traits. Note that we also included 2-way and 3-way interaction terms of observational behavior in Equation (8). The other type was participants’ personality traits. Those two types of predictor variables were assumed to have fixed effects, while there were two random effects, one for participants ( $r_{i}^{R}$) and the other for faces ( $r_{j}^{R}$).

(7) $$Ordered\;Logistic\;\left(k\;|\;\eta ,\;c\right)=\;\left\{ \begin{array}{ccc} 1-logit^{-1}\left(\eta -c_{1}\right) & & \;if\;k=1\;\;\\ logit^{-1}\left(\eta -c_{k-1}\right)-logit^{-1}\left(\eta -c_{k}\right) & & \;\;\;\;\;\;\;if1<k<K\\ logit^{-1}\left(\eta -c_{K}\right) & & if\;k=K \end{array}\right.\;$$

(8) $$\begin{array}{c} Y_{ij}∼Ordered\;Logistic\left(\sum_{g=1}^{3}\beta_{g}^{RG}G_{ijg}+\sum_{l=1}^{5}\beta_{l}^{RP}p_{il}+r_{i}^{R}+r_{j}^{R},c\right) \end{array}$$

The Rstan package was used for the parameter estimations (33; 25; 49). All parameters appear in Equations (1) to (8) were estimated simultaneously. The prior distribution of fixed effects followed the normal distribution with a mean of 0 and a variance of 100, and the prior distribution of random effects followed the gamma distribution ( α = 10, β = 10). Each model was executed with the default stan hyperparameter values; the number of chains was 4; the number of thins was 1; the number of iteration steps was 2000; and the number of warm-up steps was 1000. The number of MCMC samples obtained was 4000.

In order to confirm whether the MCMC estimations had converged, we calculated Rhat ( $\hat{R}$) for each parameter, which is often used as a judgment index for convergence. As in typical MCMC estimation, we consider estimations had “convergence” when the number of chains was greater than or equal to three and $\hat{R}$ is less than 1.1 for all parameters. Based on these criteria, all parameter estimation was confirmed converged.

## Experiment 1

In Experiment 1, we conducted simple impression inference tasks asking participants to freely observe facial images. We recorded participants’ eye movements using an eye-tracking device. The data correspond to where and how long participants looked at particular areas of faces while observing facial images in impression inference tasks. In addition, we collected data on participants’ personality traits using a questionnaire. We then analyzed data to examine how participants’ personality traits, eye movements, and impression inferences relate to each other. All participants provided a written, signed informed consent. This experiment was reviewed and authorized by Chiba University Research Review Institute (authorization #202012-1). All methods were performed in accordance with the relevant guidelines and regulations.

### Participants

The sample size for this study was set at a minimum of 30 participants, based on prior research (7; 41). Thirty-four students (undergraduate and graduate) from Chiba University with normal or corrected-to-normal vision participated in Experiment 1. Among them, 18 were female and 16 were male, with an average age of 22.1 years (SD = 3.3). Participants included both Japanese and Chinese students who had resided in Japan for more than 5 years and had no difficulty with the Japanese language. All participants were rewarded with a 500-yen gift certificate for their participation in the experiment.

### Experimental and Data Analysis Design

In this experiment, data were collected on participants’ impression ratings of stimulus images, eye movements in response to stimuli (eyes, nose, mouth), and participants’ personality traits (Big Five). The recording of eye movements using an eye tracker covers the period from the start to the end of the experiment, but only the data collected while observing the stimuli will be were analyzed. When eye movements were used as the dependent variable, participants’ personality traits were used as explanatory variables. When impression ratings were used as the dependent variable, participants’ eye movements and personality traits were used as explanatory variables. Random effects were set for both stimulus images and participants. For further details, please refer to ‘General Methods’. To avoid confusion when describing the results, impression ratings will be written out in full, while personality traits will be abbreviated (AGR for agreeableness, CON for conscientiousness, EXT for extraversion, NEU for neuroticism, and OPE for openness).

### Procedure

There was a total of 50 trials in Experiment 1. Each trial started with a brief description of a randomly selected personality trait to be rated. When participants click a mouse to confirm the description, then a fixation marker (i.e., “+”) was presented at the center of the monitor for one second, followed by a randomly selected face (within a corresponding personality set) for 3 seconds. After observing each face, participants were asked to rate the face on the impression inference asked at the beginning of the trial using a 7-point Likert scale. In each trial, participants rated a single face for its single impression. Please refer to Appendix 1 in the public database (https://osf.io/bn93u) for an illustrative diagram of the experimental procedure.

After completing all impression inference tasks, participants were asked to complete the Japanese version of Ten Item Personality Inventory (TIPI) to measure participants’ five personality traits, namely Agreeableness, Conscientiousness, Extraversion, Neuroticism, and Openness to experience Oshio et al. (39). TIPI-J provides adequate measurements of the Big Five personality traits as compared to the Japanese version Revised NEO Personality Inventory (NEO-PI-R-J), which used a much larger number of items to measure personalities (REF).

## Results of Experiment 1

Our model comprises two sub-models. The first consists of ordinal logistic models, with the impression inference ratings as the dependent variables. The second involves zero-inflated beta distribution (ZIB) models, with eye movement data as the dependent variable. For the sake of simplicity, we will describe the results of each sub-model separately.

Table 1 shows the results of the ZIB models (the results of all analyses, including data, are available at OSF https://osf.io/bn93u). Only the posterior distributions of parameters whose 95% highest density interval (HDI) — which is generally considered to be analogous to a 95% confidence interval in the frequentist approach — did not include 0 are shown in these tables. We state that an effect was ‘significant’ whenever the HDI of the corresponding posterior distribution did not include 0. HDI is a type of confidence interval in Bayesian statistics, representing the range of values with the highest probability density in the posterior distribution, often expressed as a 95% interval. Unlike a single point estimate, HDI provides a more informative view of the uncertainty associated with the results (31; 47).

**Table 1.** Significant Predictors in ZIB models in Experiment 1.

					95% HDI	
Model	Impression	Area	Predictor	Mean	Lower	Upper
Bernoulli	Agreeableness	Eye	EXT	0.806	0.109	1.569
		Mouth	NEU	2.594	0.677	4.763
		Nose	OPE	-0.664	-1.160	-0.237
	Conscientiousness	Eye	OPE	-0.867	-1.683	-0.075
		Mouth	NEU	0.913	0.051	1.793
		Nose	OPE	-0.889	-1.409	-0.426
	Extraversion	Eye	EXT	0.827	0.131	1.594
			OPE	-1.125	-2.076	-0.292
		Mouth	AGR	0.941	0.177	1.734
		Nose	OPE	-0.843	-1.435	-0.278
	Neuroticism	Eye	NEU	1.210	0.066	2.506
		Mouth	NEU	1.116	0.227	2.157
		Nose	OPE	-0.800	-1.420	-0.162
	Openness	Eye	OPE	-1.451	-2.572	-0.380
		Nose	OPE	-0.677	-1.213	-0.132
Beta	Agreeableness	Nose	AGR	-0.282	-0.573	-0.001
	Extraversion	Nose	AGR	-0.332	-0.599	-0.073
			NEU	0.216	0.005	0.447
	Neuroticism	Nose	OPE	-0.425	-0.764	-0.095

As shown in Table 1, eye movements were influenced by participants’ personality traits. The results can be summarized as follows:

When assessing Agreeableness, we found that individuals high in EXT tended to look at the eyes. Additionally, those high in NEU tended to look at the mouth, and those high in OPE tended not to look at the nose.

When assessing Conscientiousness, individuals high in NEU tended to look at the mouth. Conversely, those high in OPE tended not to look at the eyes or the nose.

When assessing Extraversion, individuals high in EXT tended to look at the eyes. Moreover, those high in OPE tended not to look at the eyes or the nose, and those high in AGR tended to look at the mouth.

When assessing Neuroticism, individuals high in NEU tended to look at both the eyes and mouth. We also found that those high in OPE tended not to look at the nose.

When assessing Openness, individuals high in OPE tended not to look at the eyes or the nose.

**Figure 4.**
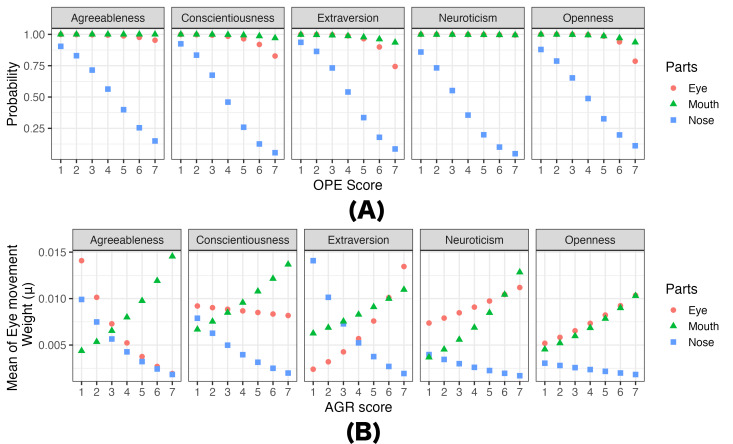
Predicted (A) probability of eye movement (eyes, nose, mouth) and (B) gaze weight as participants’ personality trait of (A)OPE And (B) AGR changes from 1 to 7 in Experiment 1. Note. In the prediction model, non-targeted personality traits (e.g., AGR, CON, EXT, and NEU in predicting the effect of OPE) were fixed at the mean values of each personality trait.

Since it is challenging to conceptualize the effects of personality traits on eye movements with the numbers provided in Table1, we visualize them for illustrative purposes in Figure 4. We refer to these models as predictive models of personality traits on eye movements. Each of these models demonstrates the effect of one personality trait (i.e., a maximum a posteriori probability estimate) while holding other personality traits constant. We chose mean values of personality traits as the constants in predictive models. Note that these results are based on simulations and include non-significant findings. (All predictive results are available on OSF at https://osf.io/bn93u.)

Figure 4 (A) shows relationships between the probabilities of looking at particular areas of faces and observers’ OPE scores while holding the scores of other personality traits constants at their means. It shows that the probabilities of looking at the nose significantly (cf. Table 1) decrease as observers’ OPE scores increase, regardless of inference tasks. Figure 4 (B) shows relationships between the gaze weights (Beta model’s μ) for particular areas of faces and observers’ AGR scores while holding the scores of other personality traits constants at their means. It shows that the gaze weight of the eyes increases as observers’ AGR score increases while that of the nose decreases as the score increases when they would rate faces’ Extraversion (cf. Table 1).

We counted the numbers of significant random effects for both participants ( $r_{ik}^{Z}$, $r_{ik}^{B}$) and faces ( $r_{jk}^{Z}$, $r_{jk}^{B}$) in the ZIB models, where ‘significant’ means that the random effects’ 95% HDI did not include 0. Table 2 summarizes the proportions of significant random effects. Overall, approximately 7.8% of participant random effects and 2.0% of face random effects in the Bernoulli component of the ZIB models were significant. For the Beta component, these proportions were 12.8% for participants and 0.6% for faces. The generally low proportion of significant facial random effects in the ZIB models suggests that specific facial features do not necessarily induce specific eye movements. This indicates that in tasks involving the rating of facial impressions, the influence of participants’ characteristics (such as personality traits and observational behavior) may be more significant.

**Table 2.** Proportions of significant random effects in ZIB models in Experiment 1.

Model	Impression	Subject (%)	Face (%)	Subject or Face (%)
Bernoulli	Agreeableness	7.8	6.7	7.6
	Conscientiousness	8.1	0	6.2
	Extraversion	8.8	3.3	7.6
	Neuroticism	8.3	0	6.4
	Openness	6.1	0	4.7
	Mean	7.8	2.0	6.5
Beta	Agreeableness	17.6	0	13.6
	Conscientiousness	10.1	0	7.7
	Extraversion	11.8	3.3	9.8
	Neuroticism	10.4	0	7.9
	Openness	14.1	0	10.9
	Mean	12.8	0.6	10

Table 3 presents the results of the ordered logistic models. These results indicate that participants with high levels of AGR tended to infer that the faces were more agreeable. Additionally, it was found that faces were perceived as less conscientious when participants focused on the nose. These findings suggest that participants’ personality traits and observational behaviors exert some influence on the personality impression inferences made about faces. However, the magnitude of these effects is weak.

**Table 3.** Significant Predictors in Ordered logistic model in Experiment 1.

			95% HDI	
Impression	Predictor	Mean	Lower	Upper
Agreeableness	AGR	0.403	0.013	0.776
Conscientiousness	Nose	-35.861	-65.121	-5.908

Subsequently, we calculated the number of significant random effects for participants and facial stimuli in ordered logistic models. Table 4 summarizes the proportions of significant random effects. Overall, approximately 3.6% of participant random effects and 44.0% of facial random effects were significant. It is important to note that in the analysis, the observer’s personality traits and observational behavior were included as explanatory variables, meaning the ’participant random effects’ mentioned here do not encompass these effects. The substantial proportion of significant random effects for faces suggests that specific facial features indeed lead to certain impression inferences, supporting prior research focused on the impact of facial features on impression inferences. Conversely, the relatively low proportion of significant random effects for participants after excluding the effects of personality traits and observational behavior implies that participants’ characteristics may not be closely related to how they infer personality impressions. Additionally, the low significance of fixed effects could be due to the strong correlation between personality traits and observational behavior, as shown in Table 1, which might have obscured the relationship with impression ratings. To examine this issue further, Experiment 2 was conducted.

**Table 4.** Proportions of significant random effects in Ordered logistic model in Experiment 1.

Impression Task	Subject (%)	Face (%)	Subject or Face (%)
Agreeableness	2.9	40	11.4
Conscientiousness	0	60	14.0
Extraversion	5.8	50	15.9
Neuroticism	3.1	20	7.1
Openness	6.1	20	16.3
Mean	3.6	44	12.9

## Experiment 2

To eliminate plausible interactions between facial features and eye movements on impression inferences, we instructed participants in Experiment 2 to look at specific areas of faces during impression inference tasks. Specifically, participants were verbally instructed to focus solely on the eyes, nose, or mouth, depending on the experimental condition (referred to as “eye condition,” “nose condition,” and “mouth condition”). It should be noted that, unlike in Experiment 1, participants in Experiment 2 did not experience a condition allowing free observation. Experiment 2 was conducted as a between-subjects design, meaning that participants in, for example, the Eye condition were instructed to look only at the eyes throughout the experiment. Such restricted observations were intended to weaken the interactive effects of facial features and eye movements on impression inference. This is because, within each condition, all participants would exhibit the same or similar eye movements while observing the same set of facial features. Consequently, this setup allowed us to examine the effects of participants’ personality traits on impression inference, as those with different personality trait patterns observed the same facial features within each condition — an outcome not possible with free observation. Furthermore, this approach enabled us to assess the impact of eye movements on impression inference, since all participants within a condition observed the same facial features, regardless of their personality traits. All participants provided written, signed informed consent. This experiment was reviewed and authorized by Chiba University Research Review Institute (authorization #202012-1).

### Participants

The participants consisted of 103 students from Chiba University with normal or corrected-to-normal vision. They were randomly assigned to one of three experimental conditions: Eye, Nose, and Mouth. Among them, 34 participants were in the Eye condition (17 females and 17 males, mean age 21.7 years, SD = 2.9), 34 in the Nose condition (23 females and 11 males, mean age 21.3 years, SD = 2.7), and 35 in the Mouth condition (20 females and 15 males, mean age 22.8 years, SD = 4.6). All participants were rewarded with a 500-yen gift certificate for their participation in the experiment. The number of participants was determined as in Experiment 1.

The stimuli, impression inference tasks, and apparatus were identical to those of Experiment 1. The experimental procedure was also the same as in Experiment 1, except instructions reminding which condition they belonged to were presented at the beginning of each session. Please refer to Appendix 2 in the public database (https://osf.io/bn93u) for an illustrative diagram of the experimental procedure. In addition, the same data preprocessing was applied to the data obtained in Experiment 2.

Similar to Experiment 1, we excluded data where eye-tracking sampling rates fell below 60%. Following this criterion, we excluded one participant from the Openness inference task in the Eye condition, one from the Extraversion inference task in the Mouth condition, and one from every inference task in the Nose condition.

### Experimental and Data Analysis Design

In Experiment 2, data were collected on participants’ impression ratings of stimulus images and eye movements in response to stimuli (eyes, nose, mouth) under three different observation instruction conditions (focus on eyes, nose, or mouth) and participants’ personality traits. As in Experiment 1, only the data collected while observing the stimuli were included in the analyses. When eye movements under each instruction condition were used as the dependent variable, participants’ personality traits under that condition were used as explanatory variables. When impression ratings under each instruction condition were used as the dependent variable, participants’ eye movements and personality traits were used as explanatory variables. In all analyses, random effects were set for both stimulus images and participants. For further details, please refer to ‘General Methods’.

### Comparing impression inferences among experimental conditions

We compared whether participants in different experimental conditions had different impressions of the facial stimuli. To do this, we ran hierarchical Bayesian-ordered logistic models similar to those in Experiment 1. Instead of using personality traits and eye movements as predictors, we included experimental conditions (adding the Free condition, which was data obtained in Experiment 1) as the predictor variables, as shown in Equations (9) and (10). As detailed in Equations (9) and (10), we used four conditions ( $C_{id}$ in Equation (10)) as the fixed effects, with participants ( $r_{i}^{subjO}$) and facial stimuli ( $r_{j}^{picO}$) serving as the random effects.

(9) $$Ordered\;Logistic\;\left(k\;|\;\eta ,\;c\right)=\;\left\{ \begin{array}{ccc} 1-logit^{-1}\left(\eta -c_{1}\right) & & \;if\;m=1\;\;\\ logit^{-1}\left(\eta -c_{m-1}\right)-logit^{-1}\left(\eta -c_{m}\right) & & \;\;\;\;\;\;\;if1<m<M\\ logit^{-1}\left(\eta -c_{M}\right) & & if\;m=M \end{array}\right.\;$$

(10) $$\begin{array}{c} Y_{ij}∼Ordered\;Logistic\left(\sum_{d=1}^{4}\beta_{d}^{OC}C_{id}+r_{i}^{subjO}+r_{j}^{picO},c\right) \end{array}$$

### Eye movements and impression inference within conditions

**Figure 5.**
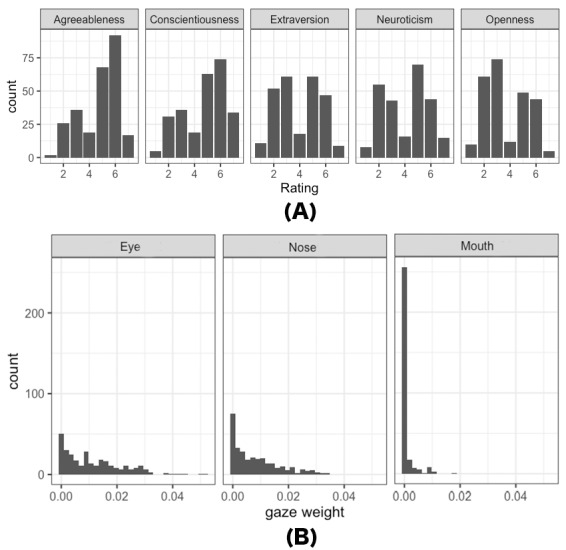
(A) Distribution of participants’ rating scores for the five impressions in the eye condition of Experiment 2, and (B) distribution of eye movements of all participants (N=34) in the eye condition of Experiment 2 when rating Extraversion for all stimuli (N=50). Note. The higher the gaze weight, the more attention is paid to the corresponding area.

Previous analyses suggested that different eye movements led to different impression inferences of faces. We now examine how these differences emerged by analyzing the relationships among participants’ personality traits, eye movements, and impression inferences within each experimental condition. Before proceeding with the analyses, we first inspected the data distributions. Figure 5 (A) displays the distributions of impression inference ratings in the Eye condition, while Figure 5 (B) shows the distributions of eye movements when rating Extraversion in the Eye condition. As in Experiment 1, we assumed that the distributions of eye movements follow a zero-inflated beta distribution (ZIB) and used ordered logistic models for impression inference. The same hierarchical Bayesian models utilized in Experiment 1 were applied in Experiment 2.

## Results of Experiment 2

### Manipulation check

**Figure 6.**
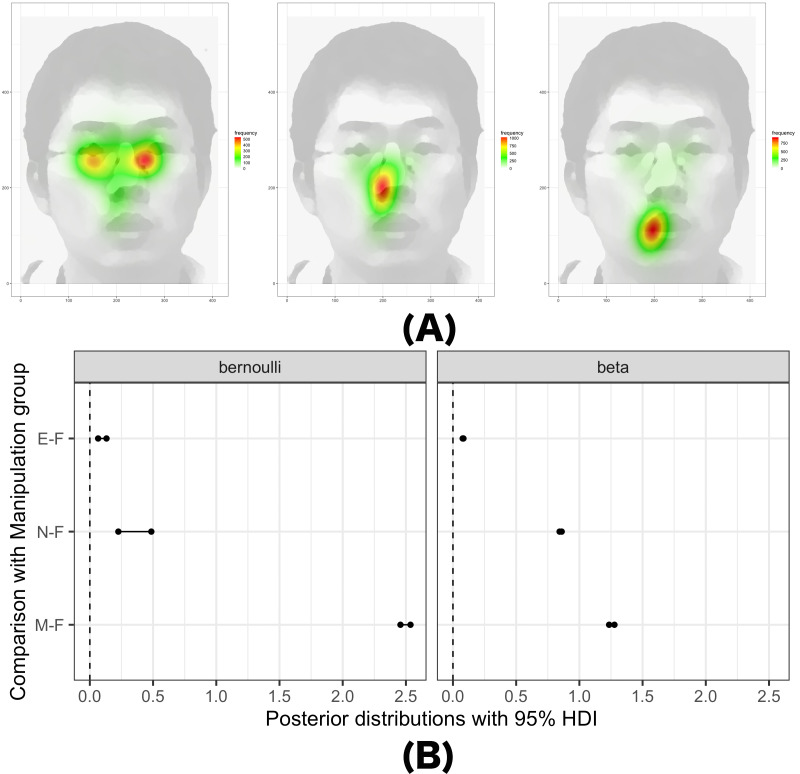
(A) Manipulation check. (B) 95% HDI of posterior distributions of pair-wise comparison between Free (control) condition and experimental conditions. Note. (A) From left to right, the heatmap of the attention of the participants whose gazes were manipulated to the eyes, nose, and mouth (These figures show the face of one of the authors as examples). (B) E-F indicates differences between Eye and Free conditions (M-F and N-F indicates differences in Mouth and Free and Nose and Free conditions, respectively). The further to the right from the dashed line, the more eye movements occurred toward the corresponding area in the experimental conditions.

In order to verify whether the gaze manipulation worked as planned, we first created a heatmap of observational behavior for each experimental condition. As shown in Figure 6 (A), the eye movements in each condition were properly concentrated on the eyes, nose, or mouth, depending on the experimental conditions. Additionally, we used a ZIB model to quantitatively test whether the observational behavior of each condition group was properly manipulated. The ZIB model used here was almost identical to that of Experiment 1, except that its predictors were experimental conditions rather than personality traits. Figure 6 (B) shows the 95% HDI of posterior distributions of pairwise comparisons between experimental conditions and the control condition, which was the data obtained in Experiment 1 where participants observed the faces without any restrictions. We refer to this control condition as the Free condition, because participants in this condition were able to observe faces without any restrictions. The further to the right from the dashed line, the more eye movements occurred toward the corresponding area in the experimental conditions (i.e., instructed to focus on the eyes, mouth, or nose). For example, participants in the Eye condition (indicated as ‘E-F’ on the y-axis) observed the eyes significantly more than those in the Free condition. The same was true for other conditions, suggesting that our manipulation of eye movements worked as we intended.

### Personality traits and Eye movements

Table 5 shows significant personality predictors for eye movements in the Eye and Nose conditions. Our manipulation check indicated that participants generally looked at the facial areas they were instructed to focus on. However, significant personality effects on eye movements were found in the Eye and Nose conditions, with no significant effect observed in the Mouth condition.

**Eye condition**: When inferring faces’ Conscientiousness, participants with a higher degree of OPE tended to focus on the eyes. Conversely, those with a higher degree of AGR tended not to look at the nose in the Eye condition.

**Nose condition**: While inferring the Agreeableness of facial stimuli, participants with a higher degree of AGR tended not to focus on the nose. In the assessment of Extraversion, participants with a higher degree of OPE were more inclined to look at the eyes, whereas those with a higher degree of EXT preferred to look at the mouth. In the assessment of Neuroticism, participants with a higher degree of both AGR and OPE tended not to look at the nose. Furthermore, those with a higher degree of EXT showed a tendency to focus on both the nose and mouth.

**Mouth condition**: Although no significant trend was observed in the Bernoulli model, the Beta model indicated that participants with a high degree of AGR tended to look at the nose when inferring Neuroticism.

**Figure 7.**
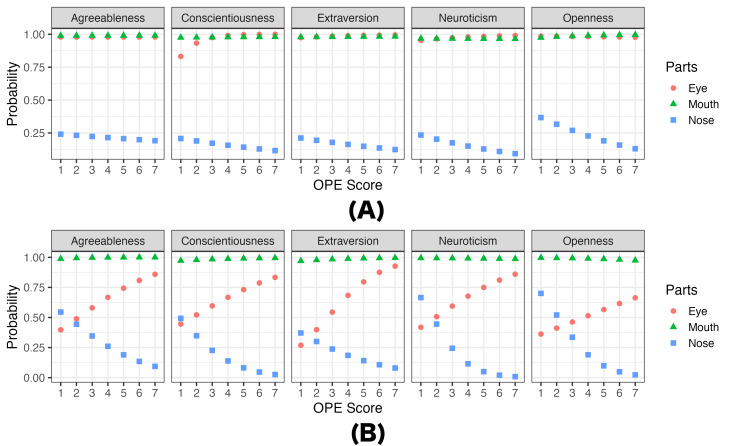
Predicted probabilities of eye movements (eyes, nose, mouth) in Experiment 2 as participants’ OPE personality trait changes from 1 to 7 in (A) the eye condition and (B) the nose condition. Note. In the prediction model, non-targeted personality traits (e.g., AGR, CON, EXT, and NEU in predicting the effect of OPE) were fixed at the mean values of each personality trait.

**Figure 8.**
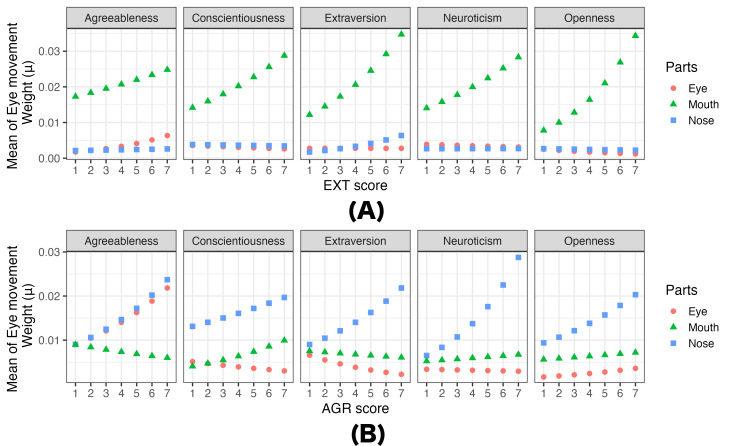
Predicted the gaze weight to each part (eyes, nose, mouth) in Experiment 2 as participants’ (A)EXT And (B)AGR personality trait changes from 1 to 7 in (A) the nose condition and (B) the mouth condition. Note. In the prediction model, non-targeted personality traits (e.g. AGR, CON, EXT, and NEU in predicting the effect of OPE) were fixed at the mean values of each personality trait.

As in Experiment 1, predictive models of eye movements using personality traits were constructed based on the estimated parameters. Figures 7 and 8 illustrate the probability of looking at each area (Bernoulli) and the gaze weight (Beta) for the eyes, nose, and mouth, respectively. These were selected as examples because they are items with a relatively large number of significant differences, as indicated by the results in Table 5. It is important to note that these models are provided for illustrative purposes, and some models may contain non-significant results. (All predictive results are accessible on OSF at https://osf.io/bn93u.)

Figure 7 (A) shows relationships between the probabilities of looking at particular areas of faces and observers’ OPE scores while holding the scores of other personality traits constant in eye condition. It indicates that participants were predicted to briefly look at mouth despite being instructed to look at only the eyes. Figure 7 (B) shows relationships between the probabilities of looking at particular areas of faces and observers’ OPE scores while holding the scores of other personality traits constant in nose condition. The results indicates as OPE score increases participants became less likely to look at the nose despite being instructed to do so. All conditions exhibited the same trend across all impression rating tasks. For example, in the eye condition, those higher in AGR tended not to look at the nose.

Figure 8 shows the predicted results of gaze weight influenced by the observer’s EXT (nose condition), and NEU (mouth condition) scores. It was found that participants with a high degree of EXT tended to look at the mouth more when rating Extraversion in the nose condition.

**Table 5.** Significant Predictors in ZIB models in Experiment 2.

						95% HDI	
Model	Condition	Impression	Area	Predictor	Mean	Lower	Upper
Bernoulli	Eye	Consc.	Eye	OPE	1.036	0.278	1.868
			Nose	AGR	-0.505	-0.910	-0.103
	Nose	Agree.	Nose	AGR	-0.471	-0.918	-0.028
		Extra.	Eye	OPE	0.589	0.005	1.136
			Mouth	EXT	0.901	0.202	1.615
			Nose	AGR	-0.473	-0.980	-0.008
		Neuro.	Nose	AGR	-0.695	-1.305	-0.085
				EXT	0.687	0.132	1.292
				OPE	-0.908	-1.624	-0.134
			Mouth	EXT	1.069	0.244	1.935
		Open.	Nose	OPE	-0.765	-1.425	-0.022
Beta	Mouth	Neuro.	Nose	AGR	0.251	0.002	0.492
	Nose	Extra.	Mouth	EXT	0.178	0.002	0.354
		Open.	Mouth	EXT	0.251	0.044	0.471

Note. Agree. = Agreeableness, Consc. = Conscientiousness, Extra. = Extraversion, Neuro. = Neuroticism, Open. = Openness.

### Impression inferences

Table 6 summarizes the significant effects of participants’ characteristics on impression inferences for each condition. There were numerous significant effects of participants’ personality traits on impression inferences within the manipulation conditions. Specifically, when participants were instructed to focus on the eyes, the influence of personality traits on impressions became readily apparent. For example, individuals with a higher degree of NEU were more likely to rate the facial stimuli as lower in Agreeableness and Extraversion after being instructed to look at the eyes. Similarly, those with a higher degree of AGR and CON tended to rate the faces as lower in Agreeableness. In the Nose condition, participants with a higher degree of OPE were inclined to give higher Agreeableness ratings to the faces. In the Mouth condition, those with a higher degree of OPE tended to rate the faces higher in Openness.

**Table 6.** Significant Predictors in Ordered logistic model in Experiment 2.

				95% HDI	
Condition	Impression	Predictor	Mean	Lower	Upper
Eye	Agreeableness	AGR	-0.267	-0.514	-0.018
		CON	-0.274	-0.542	-0.001
		NEU	-0.272	-0.524	-0.030
	Extraversion	NEU	-0.348	-0.684	-0.036
Nose	Agreeableness	OPE	0.473	0.142	0.778
Mouth	Conscientiousness	NEU	0.339	0.008	0.696
		Nose	28.335	2.117	52.099
	Openness	OPE	0.387	0.040	0.773

### Result of comparing impression inferences among experimental conditions

**Figure 9.**
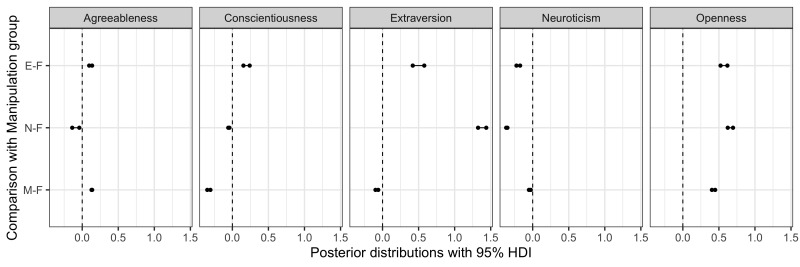
95% HDI of posterior distributions of pair-wise comparison between Free (control) condition and experimental conditions in ratings. Note. E-F indicates differences between Eye and Free conditions (M-F and N-F indicates differences in Mouth and Free and Nose and Free conditions, respectively). The further to the right from the dashed line, the higher the ratings under the experimental conditions, and the further to the left from the dashed line, the higher the evaluation results under Free observation condition.

Figure 9 presents pairwise comparisons between the control and three experimental conditions, demonstrating that participants in different experimental conditions indeed had varying impressions of the identical facial stimuli. Specifically, even when observing the same faces, participants formed distinct impressions based on instructions to focus on specific areas of the faces. This effect was most pronounced in the inference of the stimuli’s Extraversion ratings — the most significant degree of change was observed in conditions where observation behavior towards the nose was manipulated. An interesting observation was that when observation behavior towards the eyes was manipulated, all impressions of the faces shifted positively (with higher ratings, except for the Neuroticism rating), indicating that the overall impression of the person became more positive. As a comparison, traditional statistical analysis using the ordinal package in R was also conducted. However, due to the inability to account for complex individual differences and hierarchical prior information, significant differences were only observed in some aspects of Conscientiousness, Extraversion, and Openness. These results suggest that the advantages of Bayesian statistics were sufficiently reflected.

## Discussion

The present research explored the direct and indirect effects of participants’ personality traits on the impression inference of human faces. Several potential indirect effects of observers’ personalities on impression inferences were investigated in this study. We assumed that observers’ personality traits influence how they observe faces, subsequently affecting impression inferences. Observational behavior data were collected using an eye tracker, processed with a Gaussian filter, and normalized for each area of interest (i.e., eyes, nose, and mouth). The Japanese version of the Ten-Item Personality Inventory was used to measure participants’ Big Five personality traits, which were also the traits participants rated for the faces in the experiments. The results of Experiment 1 revealed relationships between participants’ personality traits and eye movements, indicating that individuals with specific personality traits tended to focus on particular areas of the faces. However, weak relationships were found between participants’ personality traits and impression inferences, as well as between eye movements and impression inferences. This suggests that participants’ personalities did not significantly influence how faces were perceived (i.e., impressions). Detailed model analyses indicated that while individual differences among participants had a more substantial impact on observational behaviors than those of faces, the effects of individual differences among faces on impression inference were more pronounced than those of participants. In essence, in the process of inferring faces, facial features strongly influenced the resulting impressions, whereas participants’ individual differences did not. Conversely, eye movements were not influenced by specific facial features but rather by participants’ personality traits. This finding aligns with previous research in other domains that has observed the effect of personality on eye movement patterns. However, to the best of our knowledge, it is a novel finding that personality traits do not directly influence impression ratings but do so indirectly through eye movements. To further verify this relationship, additional experiments will be necessary in future research.

We hypothesized that the weak effects of participants’ characteristics on impression inferences in Experiment 1 might have been caused by a potential interactive effect between facial features and participants’ eye movements. Experiment 2 was conducted to control this potential interaction on impression inferences by manipulating participants’ eye movements. Specifically, we asked participants to focus on either the eyes, nose, or mouth during impression inference tasks. The results of the manipulation check confirmed that participants mostly looked at the areas where they were instructed to look. Our analyses suggested that looking at different areas of faces led to different impression inferences. This implies that simply focusing on different areas of faces, regardless of individual differences in facial features, results in different impressions. These findings suggest, for example, that regardless of the sizes and shapes of the eyes, just looking at the eyes makes people perceive a face as having a higher degree of OPE.

This interpretation may seem counterintuitive, but for the following reasons, it may be reasonably sound. A study exploring the relationship between self-control and mindset indicated that individuals demonstrate more self-control, manifested as gaze control, during abstract thinking tasks compared to concrete ones (30). In other words, it is more challenging for humans to engage in concrete thinking while controlling their gaze, and vice versa. In Experiment 2, we manipulated participants’ observational behavior, limiting their self-control, which subsequently altered their thinking mindsets compared to those in Experiment 1. In Experiment 2, the study was conducted with eye control restrictions, but some participants were observed to have eye movements that did not align with the instructions. Although this did not significantly impact the experimental results, we believe there might be an underlying cause for this behavior. We intend to investigate this intriguing cause in future studies. Moreover, it is well-known that different areas of the face are associated with distinct emotions. The upper area of the face is linked to anger, fear, surprise, and sadness, while the lower area is associated with disgust and happiness (15). Looking at specific areas of the face as instructed reminded participants of particular emotional states, resulting in different impression inferences. Similarly, looking at specific areas reminded them of particular actions. For instance, focusing on the mouth made participants imagine “talking,” leading them to infer that the face had a higher degree of Openness. Another potential explanation is that it was not looking at specific areas that led to different impressions, but rather looking at the face freely canceled out the effects of facial areas, resulting in “average” impression inferences.

Our detailed analyses revealed some effects of personality traits on impression inferences when eye movements were manipulated. It appeared that the effects of observational behavior and personality traits, which typically influence each other alternately, were evident with gaze manipulation. Simultaneously, in comparison to Experiment 1, the relationships between personality traits and observational behavior were weakened in Experiment 2. This is likely due to gaze manipulation restricting eye movements. Nevertheless, the relationships between observational behavior and personality traits seemed to persist even when the gaze was manipulated.

### Conclusion and Future Work

The present research explored the direct and indirect effects of participants’ personality traits on impression inference of human faces. Experiment 1 revealed relationships between participants’ personality traits and eye movements, indicating that individuals with specific traits focused on particular facial areas. However, weak correlations were found between personality traits and impression inferences, suggesting that participants’ personalities did not significantly influence how faces were perceived. Experiment 2 controlled for potential interactions between facial features and eye movements by manipulating participants’ gaze. The results suggested that focusing on different facial areas led to distinct impression inferences, regardless of individual facial differences. Relationships between personality traits and impression inferences became clearer when eye movements were manipulated, while the link between personality traits and observational behavior weakened. These findings imply that participants’ personality traits influence face perception primarily through their observational behaviors, emphasizing the need for future research on the interplay between gaze control and mindset.

On the other hand, this study has several limitations. First, there are individual differences in the stimulus images. Although previous research has validated this, even with neutral expressions, perceptions may vary between individuals. How to completely eliminate this influence remains a challenge for future research. Furthermore, previous studies have shown that impressions can be formed in extremely short periods of time, so the effect of the order in which the face’s parts are observed should be considered in future research. Using the time-series data analysis, which is not used in the present study, could yield richer and more robust results. Additionally, not only eye movements and personality traits but also changes in participants’ emotions and moods could influence impression inferences. Given that the same individuals showed different eye movements across various impression inference tasks (such as inferring openness and neuroticism), careful consideration of stimuli, tasks, and experimental design is needed for future experiments.

### Data Availability Statement

Our datasets, stan model and R code generated during this study are available on OSF at https://osf.io/bn93u/. The stimulus images can also be shared for research purposes. If needed, please contact the authors.

### Funding

This work was supported by Japan Society for Promotion of Science Kakenhi (URL: https://www.jsps.go.jp/english/): grant numbers 16H028354 to Toshihiko Matsuka. The funder had no role in study design, data collection and analysis, decision to publish, or preparation of the manuscript.

### Ethics and Conflict of Interest

The authors declares that the contents of the article are in agreement with the ethics described in http://biblio.unibe.ch/portale/elibrary/BOP/jemr/ethics.html and that there is no conflict of interest regarding the publication of this paper.
